# Exploring rare locations of hydatid disease: a retrospective case series

**DOI:** 10.1186/s12893-024-02443-x

**Published:** 2024-05-17

**Authors:** Cherif Mona, Mesbahi Meryam, Khedhiri Nizar, Benzarti Yazid, Amal Amara, Zaafouri Haithem, Hadded Dhafer, Ben Maamer Anis

**Affiliations:** Surgical Department, Habib Thameur Hospital, Tunis, Tunisia

**Keywords:** Parasitic infection, Hydatid disease, Surgery

## Abstract

**Introduction:**

Hydatid disease, endemic in Mediterranean countries, primarily affects the liver, but can manifest in diverse organs. Non-hepatic and non-pulmonary cysts often pose diagnostic challenges. This study examines patients with hydatid cysts in atypical locations.

**Methods and results:**

From 2013 to 2020, our center treated 250 echinococcosis patients, among whom 11 cases (4.4%) with hydatid disease in uncommon sites were retrospectively reviewed. The distribution of unusual cyst locations and their clinical implications are discussed.

**Conclusion:**

Diagnosing hydatid cysts in uncommon locations is a formidable challenge. Surgeons should always contemplate the prospect of an unconventional cyst location when encountering patients with cystic masses in endemic regions. Failing to consider this possibility could lead to unfavorable outcomes.

## Introduction

Hydatid disease (HD) is a parasitic infection caused by the larval stage of the Taenia Echinococcus parasite. It is considered an endemic condition in regions such as the Middle East, Africa, Australia, Turkey, India, and parts of Southern Europe. Humans inadvertently become intermediate hosts by ingesting Taenia eggs. Echinococcus granulosus infestation in humans predominantly affects the liver (55–70%) followed by the lungs (18–35%) [1]. although less common, hydatid cysts can occur in unusual sites, constituting around 8–10% of reported cases in the literature [1]. These uncommon locations include the spleen, kidney, peritoneal cavity, skin, and muscles which incidence is about 2% each, and in rarer instances, the incidence of HD is about 1% each including the heart, brain, vertebral column, ovaries, pancreas, gallbladder, thyroid gland, breast, and bones [1].

The objective of this study is to highlight the occurrence and management of hydatid cysts in atypical sites, contributing to a better understanding of this aspect.

## Materials and methods

A retrospective case series study was carried out in our Surgical Department between 2013 and 2020. We reviewed 11 cases of hydatid disease in uncommon locations among 250 echinococcosis patients treated during this period. Data on clinical presentation, imaging findings, and treatment outcomes were analyzed. Ethical clearance was obtained for the study. All data used in this study were collected by a principal doctor. The personal information of patients was protected following the Chinese law, and only the case numbers were provided for identification.

## Results

Among the 11 patients with hydatid cysts in uncommon sites, distinctive distributions were observed. Extrahepatic cysts were predominant, with splenic involvement in 6 cases and pelvic cysts in 3 cases. Other rare locations included gluteal and spermatic cord cysts. All patients presented with symptoms, and diagnostic imaging played a crucial role in confirming the diagnosis (Table 1 summarizes all cases). Surgical management was employed, with total cystectomy being the preferred approach. Primary involvement of unusual locations was seen in 10 patients. All our patients were symptomatic. The Echinococcus antibody Immunoglobulin G (IgG) assay which is an enzyme-linked immunosorbent assay (ELISA) based qualitative detection of IgG antibodies was used in all patients. A CT scan was performed in 11 cases. The treatment course employed was combined with surgical and medical therapy. Complications during the treatment course occurred in 1 case including massive pulmonary embolism and then death. No recurrence was observed.

**Table 1:** Location, age and sex, different form of presentations, serology, imaging investigations, management and complications of all the eleven unusual cases

location	Age/Gender	Clinical features	ELISA/ biological findings	Imaging findings	Surgical management	Anatomopathological examination	Complications
splenic hydatid cyst	42/F	Left hypochondriac pain	positive	18*10*6 cm lower pole splenic hydatid cyst	Splenectomy	Splenic Hydatid disease	NO
splenic hydatid cyst	18/F	Abdominal pain	positive	15*10 cm well defined hydatid cyst upper pole spleen	Splenectomy	Splenic Hydatid disease	NO
splenic hydatid cyst	69/M	Abdominal pain	positive	a large cystic lesion replacing almost all the spleen with displacement of the stomach	albendazole 400 mg oral tablet twice per day for 3 weeks. Splenectomy	Splenic Hydatid disease	NO
splenic hydatid cyst	36/F	Abdominal pain	positive	Multiple cystic lesions in the spleen	splenectomy	Hydatid disease of the spleen	NO
splenic hydatid cyst	42/M	A mass in the left upper quadrant of the abdomen	positive	14 cm loculated cyst with many septa origination from the spleen	splenectomy	Hydatid disease of the spleen	NO
Pelvic cyst	35/F	Abdominal pain	positive	8*7 cm pelvic cyst	pericystectomy	Hydatid cyst	NO
Pelvic cyst	62/F	Chronic pelvic pain	negative	a 9 cm purely cystic mass of the left ovary classified as O-RADS 2 evocative of a serous cystadenoma	a total hysterectomy with bilateral adnexectomy	two ovarian serous cystadenomas measuring 12 cm on the left and 1.7 cm on the right and a subserous calcified hydatid cyst of the uterine fundus	NO
Gluteal cyst	71/F	A swelling in the upper outer area of her right gluteal region	Negtaive	a hydatid cyst in the gluteal region, which had affected the iliac bone, the coxo-femoral joint (Fig. 1)	total cystectomy to remove both the peritoneal and gluteal hydatid cysts involving muscle excision	Hydatid disease of the gluteal region	massive pulmonary embolism then death
Pelvic cyst	32/F	Pelvic pain	positive	a substantial cyst located retro uterine in the pelvic region, displaying intricate structures that did not exhibit enhancement with gadolinium contrast. (Fig. 2)	a peri-cystectomy	Hydatid disease was confirmed	NO
Spermatic cord cyst	40/M	discomfort in the right lower quadrant+ A tender mass in the right inguinal region	positive	inguinoscrotal hernia along with a thin-walled, cystic, anechoic mass	Cystectomy :. Upon exploration, a substantial hydatid cyst was identified protruding from the inguinal canal (Fig. 3)	a hydatid cyst was confirmed	No
splenic hydatid cyst	24/F	incidental finding during a sonography examination	positive	of a well-defined complex cystic lesion measuring approximately 7 × 5 cm at the lower pole of the splenic parenchyma	Splenectomy : A laparotomy revealing the presence of a hydatid cyst occupying the upper pole of the spleen (Fig. 4)	Hydatid disease of the spleen	NO

## Case presentation

### Case 1

**Fig. 1:**
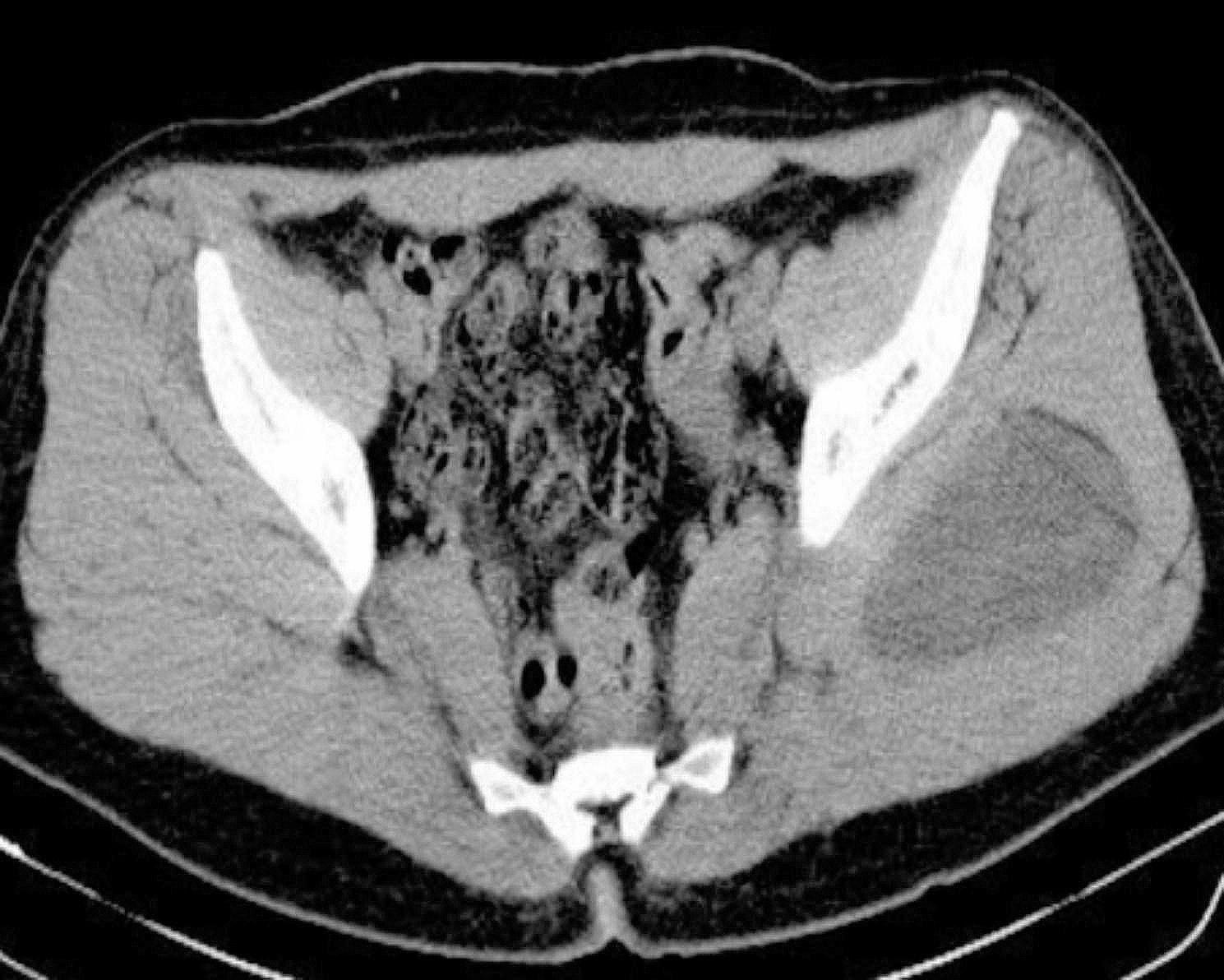
CT scan findings revealed gluteal hydatid cyst with involvement of the iliac bone, the coxo-femoral joint

A 71-year-old female visited the surgical outpatient department, reporting a primary concern of swelling in the upper outer area of her right gluteal region that had been present for the past two years. The swelling had been progressively enlarging in size. She did not experience any accompanying symptoms nor did she notice similar swellings in other parts of her body. The swelling measured approximately 10 cm, had a round shape, exhibited no tenderness, felt firm to the touch, and was immobile with poorly defined edges. CT scan results (Fig. 1) indicated the presence of a hydatid cyst in the gluteal region, which had affected the iliac bone, and the coxo-femoral joint, and also had a peritoneal component. The patient’s treatment plan involved administering albendazole in the form of 400 mg oral tablets twice daily for a duration of 3 weeks leading up to the surgery to sterilize the cyst, to decrease the chance of anaphylaxis, to decrease the tension in the cyst wall (thus reducing the risk of spillage during surgery), and to reduce the recurrence rate post-operatively. The patient underwent total cystectomy to remove both the peritoneal and gluteal hydatid cysts, involving muscle excision as well. Unfortunately, the recovery process was marred by a serious complication - a massive pulmonary embolism - and tragically, the patient passed away 18 days after the operation.

### Case2

A 32-year-old female patient presented to our department with complaints of pelvic pain. Upon physical examination, a pelvic mass was detected. An ultrasound was conducted, revealing a hydatid cyst in segment 6 of the liver, an extrahepatic hydatid cyst adjacent to the former, and a lateral retro uterine mass on the left side measuring 10 cm. This mass was suggestive of a type IV pelvic hydatid cyst.

**Fig. 2:**
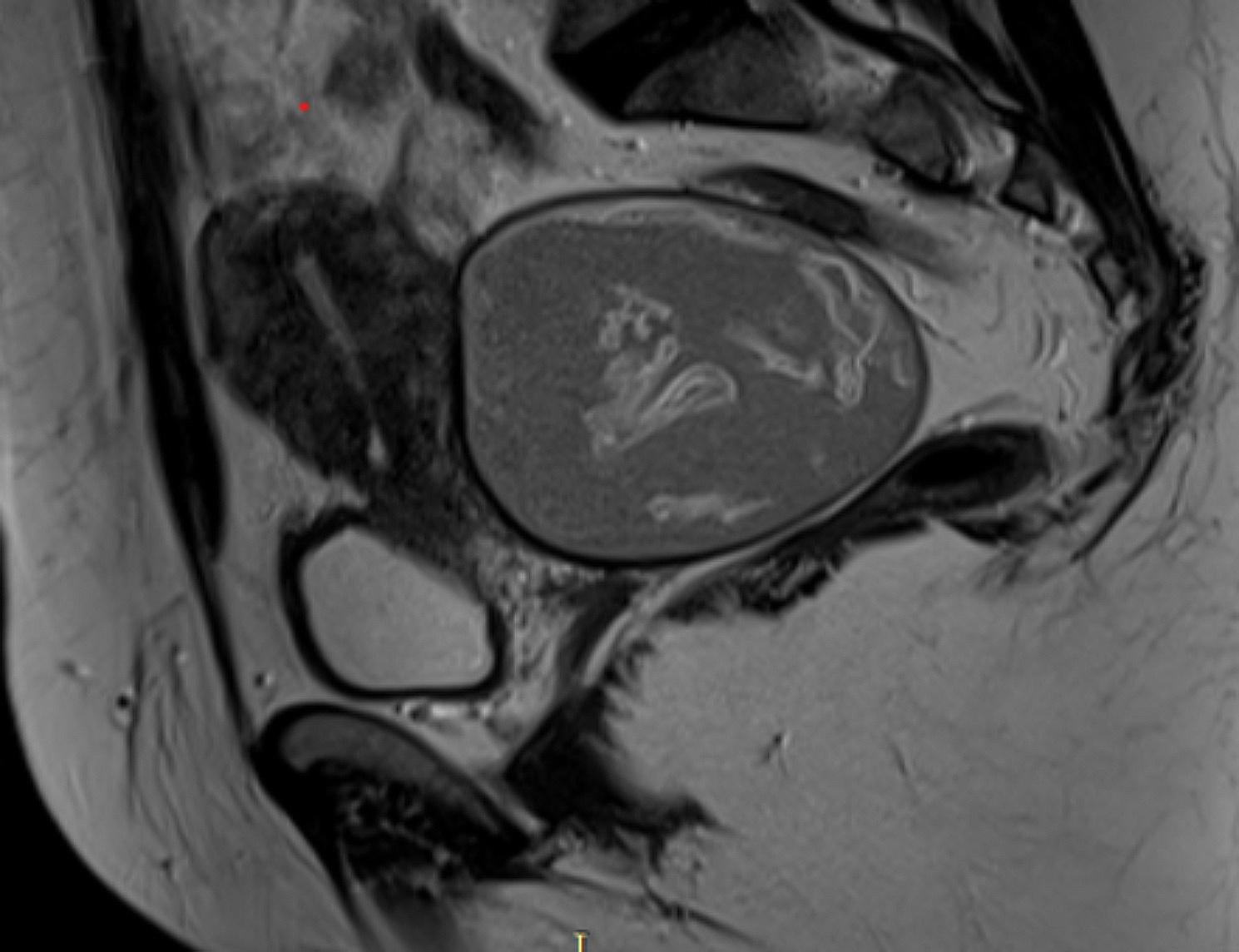
A pelvic MRI has demonstrated a voluminous pelvic cyst retro uterine, seat of serpiginous structures which not enhanced by gadolinium

A subsequent pelvic MRI (Fig. 2) exhibited a substantial cyst located retro uterine in the pelvic region, displaying intricate structures that did not exhibit enhancement with gadolinium contrast. Based on these findings, the diagnosis of a pelvic peritoneal hydatid cyst in the Douglas pouch was suspected. Consequently, the patient underwent surgery, during which a peri-cystectomy was performed. The procedure was executed without any major complications.

### Case3

A 40-year-old male patient presented with persistent discomfort in the right lower quadrant persisting for the last 2 years. During the abdominal examination, a palpable pulsatile and tender mass measuring 4 cm × 10 cm was detected in the right inguinal region. The mass exhibited mobility during coughing. Subsequent ultrasonography revealed the presence of an inguinoscrotal hernia along with a thin-walled, cystic, anechoic mass.

**Fig. 3:**
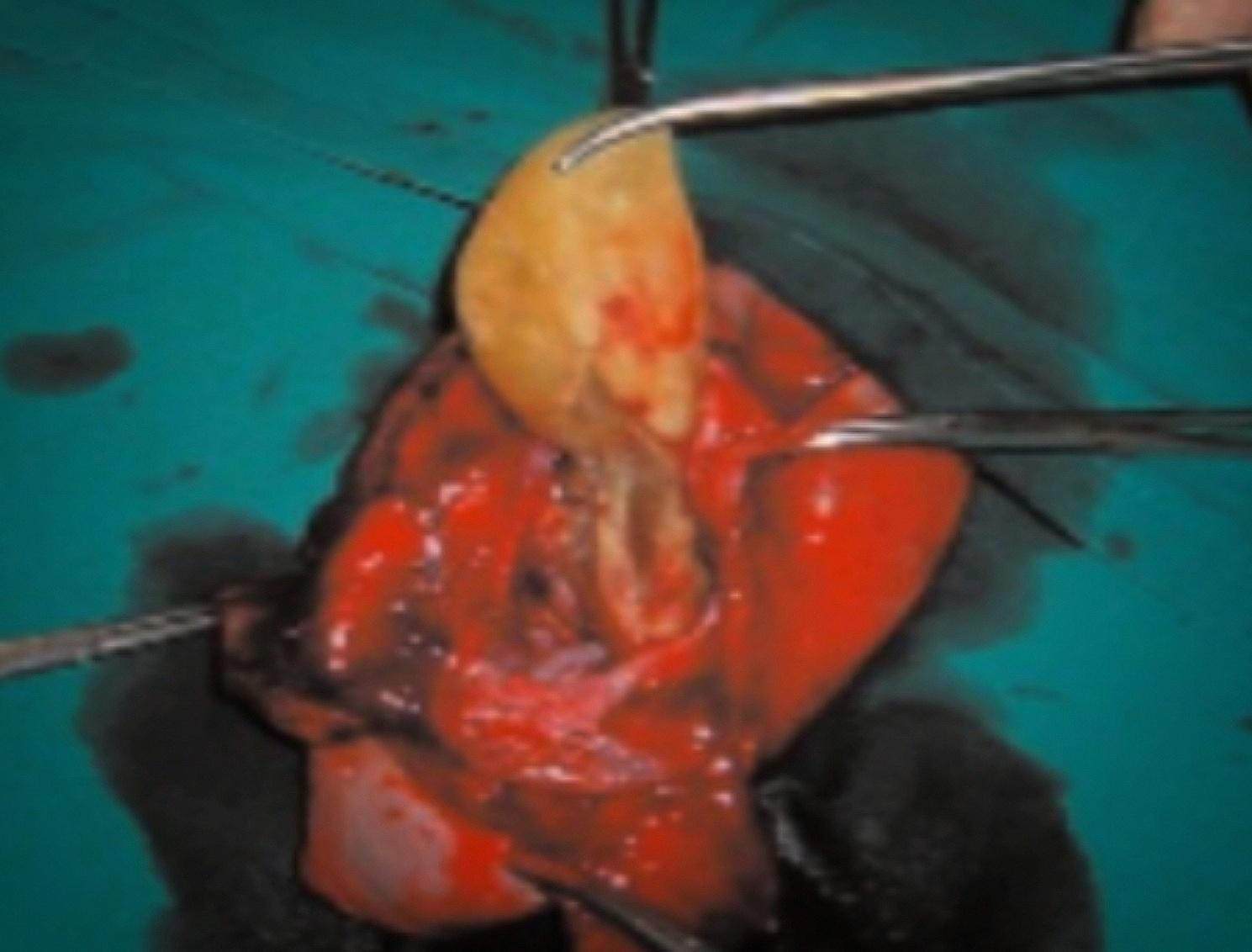
Per-operatively findings: a large firm hydatid cyst protruding from inguinal canal

The patient underwent surgery under spinal anesthesia, opting for a conventional inguinal incision as the chosen approach. Upon exploration, a substantial hydatid cyst was identified protruding from the inguinal canal (see Fig. 3). A resection of the cystic mass was performed alongside herniorrhaphy. Histological examination following the surgery confirmed the diagnosis of a hydatid cyst.

### Case4

24-year-old female patient was referred to our general surgery department following an incidental discovery during a sonography examination. The patient had no reported history of keeping pet dogs or sheep at home. During the abdominal examination, tenderness was noted, with no observed resistance or rebound tenderness. A subsequent CT scan of the abdomen confirmed the ultrasound findings, revealing a well-defined complex cystic lesion measuring approximately 7 × 5 cm at the lower pole of the splenic parenchyma. The absence of calcification suggested a hydatid cyst, with no involvement of the liver or other organs.

**Fig. 4:**
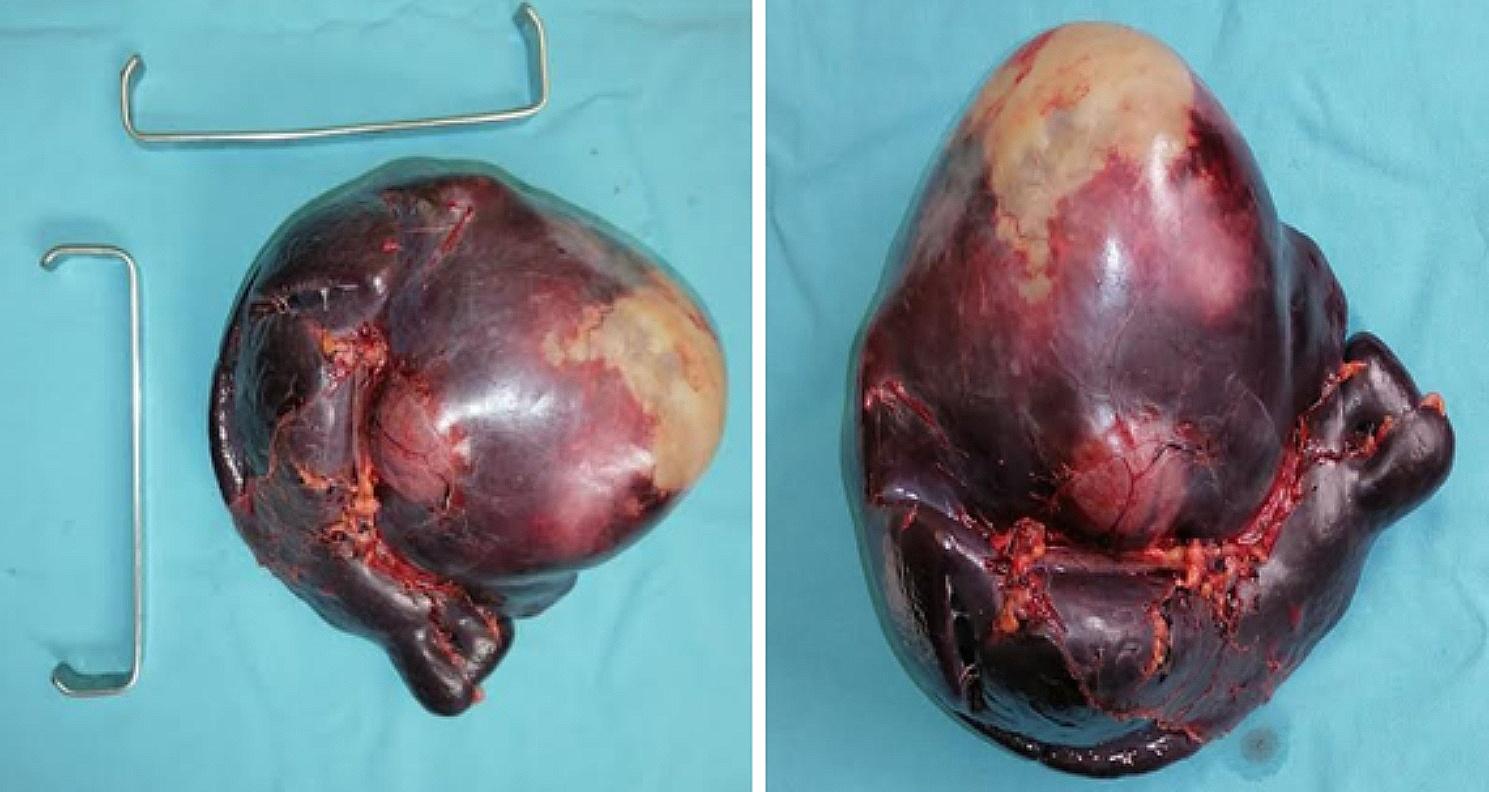
The surgical exploration revealed the presence of a hydatid cyst occupying the upper pole of the spleen

The patient’s treatment plan included the administration of albendazole in the form of 400 mg oral tablets twice daily for a duration of 3 weeks leading up to the surgery. Additionally, an Indirect Hemagglutination Assay (HAI) yielded a positive result for Echinococcus species, with a titer exceeding 160. A laparotomy was performed through a midline incision, revealing the presence of a hydatid cyst occupying the upper pole of the spleen (see Fig. 4). Subsequently, a splenectomy was carried out, and the abdominal cavity was locally irrigated with hypertonic saline solution (NaCl 20%). Both macroscopic and microscopic examinations of the specimen confirmed the presence of a hydatid cyst.

## Discussion

More than 80–90% of hydatid cysts occur in the liver, lungs, or both, However unusual locations of HD have been rarely reported [1]. Furthermore, the diagnosis of hydatid cysts in unusual sites is difficult, and differential diagnosis is always posed [2].

*The clinical symptomatology* of HD depends on the size, the site, and the extent of the lesion [2]. Extrahepatic hydatid cysts usually remain asymptomatic for years [3]. According to our results all patients presented with symptoms.

*Radiological imaging* plays including MRI, plays a crucial role in diagnosis, particularly in uncommon sites. Our study showed that, despite the usual presenting features, the locations of the cysts were.

unusual and warranted further investigation into the disease. Radiological studies of such cases also need more emphasis on commonly ignored locations such as pelvic, gluteal, and spermatic cord cysts. Ultrasonography (USG) is the first-line radiological exam, it is useful for the diagnosis by detecting cystic membranes, septa, and hydatid sand [1]. CT scan represents also a routine modality for the diagnosis of HD from another differential diagnosis, and it is useful when suspecting peritoneal seedlings. CT scan findings show a “spoke wheel” pattern a water lily sign, multiple cystic lesions, or calcification in the peritoneum [3].

*Serological tests* are useful for the diagnosis, enzyme-linked immunosorbent assay (ELISA) is the widely available serological technique to diagnose hydatid disease [3]. In our study, we did perform serology for all our patients.

Hydatid cysts can affect any organ in the body but are often located in the liver and lungs [2, 4]. Because The liver is the first sieve to encounter portal flow, most larvae are trapped and cysts begin to grow. Larvae that successfully penetrate the liver barrier proceed to the lungs, where they develop into hydatid cysts. In rare cases (10–20%), they pass through these filters and enter the systemic circulation getting into any tissues [4]. As per the existing literature, the prevalent sites included the CNS, musculoskeletal system, heart, and kidney. Nevertheless, infrequently documented sites encompass the spleen, pancreas, appendix, thyroid, salivary gland, adrenal gland, breast, and ovary [4].

Spleen localization of the hydatid cyst is uncommon, especially when the parasite primarily infects this organ [5]. This scenario arises in around 2% of instances where the parasite bypasses hepatic and pulmonary filters [6, 7]. , However, it stands as the most common extrahepatic site for hydatid cysts [8]. Diagnosing splenic hydatid cysts is typically a complex task [6]. Indeed, the symptoms are often unclear, and clinical indicators are not sufficient to establish the diagnosis [8]. Immunological findings and serology can provide valuable assistance in distinguishing between various types of splenic cysts [8]. However, imaging findings, including ultrasound (US), CT scan, and MRI, are more instrumental in confirming the diagnosis of splenic hydatid cysts [8].

Nonetheless, the peritoneal occurrence of hydatid cysts, whether originating directly or as a secondary manifestation, is a rare but noteworthy aspect of the disease, constituting a significant proportion (around 13%) [6]. Secondary peritoneal hydatid cysts typically arise when a primary cyst in the liver or spleen ruptures. Conversely, primary peritoneal hydatid cysts are observed in only 2% of all abdominal hydatid cyst cases [6].

Pelvic hydatid disease is a rare occurrence, manifesting in approximately 2.25% of cases [7]. The presence of a primary pelvic hydatid cyst is exceedingly rare, typically characterized by pressure symptoms affecting neighboring organs [5].

However, the incidence of hydatid cysts in the ovary is relatively infrequent, varying between 0.2% and 2.25% [7]. Ovarian hydatid disease often arises following the rupture of liver hydatid cysts into the peritoneal cavity, leading to the spread of the parasite within the pelvic cavity [9].

Renal hydatid cysts are likewise uncommon, accounting for only 1–3% of cases [7]. They are documented as a more frequent occurrence compared to other locations, following the liver and lungs in the literature [5]. Furthermore, retroperitoneal hydatid disease is an exceedingly rare occurrence [8].

Consequently, hydatid cysts in the pancreas are an exceptionally rare scenario, with reported occurrences ranging from 0.5 to 0.8% of cases [7]. the presence of hydatid cysts in the pancreas poses challenges both in terms of diagnosis and treatment [5].

The occurrence of a hydatid cyst in the gallbladder is highly uncommon, with an incidence of approximately 0.4% [5].

Instances of inguinal canal hydatid disease presenting as inguinal hernia are exceptionally rare, with the literature reporting only five cases [5]. Amongst our patients, only one had inguinal canal involvement.

Likewise, abdominal wall hydatid cysts are an infrequent phenomenon, with only five reported cases in the literature [5].

The management of extrahepatic hydatid cysts is contingent upon their size and location, with total cyst removal being the preferred approach [10]. For smaller cysts, medical treatment involving anti-helminthic drugs is recommended, it is also important in preventing recurrence following surgery. In our study, we did indicate it per operatively in all our patients. However, surgery becomes necessary for symptomatic and larger cysts. The preferred approach is total cyst removal [10]. In the case of splenic hydatid cysts, the management approach is subject to discussion: some advocate for conservative surgical options such as partial splenectomy or cyst drainage [8]. For abdominal wall hydatid disease, treatment entails complete excision, and in some instances, abdominal reconstruction employing prosthetic materials may be performed [8]. This study contributes to the existing literature by highlighting the clinical importance of considering hydatid disease in the differential diagnosis of cystic masses in endemic regions.

## Conclusion

Hydatid cysts can manifest in diverse organs, necessitating consideration in patients with cystic masses, especially in endemic regions. Total cyst removal remains the preferred treatment option, emphasizing the need for early diagnosis and appropriate management. Further research is warranted to explore optimal treatment strategies for hydatid disease in uncommon locations.

## Data Availability

There is no additional data available to share with the readers. The datasets used and/or analyzed during the current study are available from the corresponding author upon reasonable request.
